# Sea surface temperature dictates movement and habitat connectivity of Atlantic cod in a coastal fjord system

**DOI:** 10.1002/ece3.5453

**Published:** 2019-07-21

**Authors:** Thomas A. B. Staveley, David M. P. Jacoby, Diana Perry, Felix van der Meijs, Ingvar Lagenfelt, Mikael Cremle, Martin Gullström

**Affiliations:** ^1^ Department of Ecology, Environment and Plant Sciences Stockholm University Stockholm Sweden; ^2^ AquaBiota Water Research Stockholm Sweden; ^3^ Institute of Zoology Zoological Society of London, Regent's Park London UK; ^4^ Department of Aquatic Resources Swedish University of Agricultural Sciences Lysekil Sweden; ^5^ The County Administrative Board of Västra Götaland Gothenburg Sweden; ^6^ Department of Biological and Environmental Sciences University of Gothenburg Fiskebäckskil Sweden

**Keywords:** acoustic telemetry, coastal seascape ecology, fish movement, network analysis, seagrass habitat

## Abstract

While movements of organisms have been studied across a myriad of environments, information is often lacking regarding spatio‐seasonal patterning in complex temperate coastal systems. Highly mobile fish form an integral part of marine food webs providing linkages within and among habitats, between patches of habitats, and at different life stages. We investigated how movement, activity, and connectivity patterns of Atlantic cod (*Gadus morhua*) are influenced by dynamic environmental conditions. Movement patterns of 39 juvenile and subadult Atlantic cod were assessed in two coastal sites in the Swedish Skagerrak for 5 months. We used passive acoustic telemetry and network analysis to assess seasonal and spatial movement patterns of cod and their relationships to different environmental factors, using statistical correlations, analysis of recurrent spatial motifs, and generalized linear mixed models. Temperature, in combination with physical barriers, precludes significant connectivity (complex motifs) within the system. Sea surface temperature had a strong influence on connectivity (node strength, degree, and motif frequency), where changes from warmer summer waters to colder winter waters significantly reduced movement activity of fish. As the seasons changed, movement of fish gradually decreased from large‐scale (km) linkages in the summer to more localized movement patterns in the winter (limited to 100s m). Certain localized areas, however, were identified as important for connectivity throughout the whole study period, likely due to these multiple‐habitat areas fulfilling functions required for foraging and shelter. This study provides new knowledge regarding inshore movement dynamics of juvenile and subadult Atlantic cod that use complex, coastal fjord systems. The findings show that connectivity, seasonal patterns in particular, should be carefully considered when selecting conservation areas to promote marine stewardship.

## 1 INTRODUCTION

Measuring the movements of animals at scale is crucial for understanding linkages between organisms and their environments (Treml & Kool, 2018). In coastal seas where habitats are often patchy, studying the movement patterns of marine organisms is integral to increase our knowledge of the influence of connectivity on population dynamics and other ecological processes, for example, predation, competition, and recruitment (Olds et al., 2018; Perry, Staveley, & Gullström, 2018). Highly mobile fish, in particular, can connect different habitats or patches of habitats by transferring nutrients and energy from one location to another (Hyndes et al., 2014; Williams, Papastamatiou, Caselle, Bradley, & Jacoby, 2018). Movement patterns can be species, individual, or life‐stage specific with some species showing very clear ontogenetic shifts between habitats, from localized movements to region‐wide migrations (Andrews et al., 2009; Pittman et al., 2014).

Coastal waters and associated habitats are one of the most productive marine environments on Earth, harboring a rich suite of species, important habitats for juvenile organisms, and a plenitude of ecosystem services (Barbier et al., 2011; Beck et al., 2001; Sheaves, Baker, Nagelkerken, & Connolly, 2014). In northern temperate Europe, coastal seas are often characterized by structural forming vegetation such as seagrass and macroalgal beds. Such structurally complex habitats can offer many benefits to organisms such as nursery areas, refuge sites, and prey availability (Dahlgren et al., 2006; Jackson, Rowden, Attrill, Bossey, & Jones, 2001; Stål et al., 2008; Stål, Pihl, & Wennhage, 2007). Unvegetated soft bottoms are much less studied, particularly when focusing on movement patterns of fish (Fetterplace, Davis, Neilson, Taylor, & Knott, 2016).

The Atlantic cod (*Gadus morhua*) is an important predator in North Atlantic food webs, but also economically important as a food resource across the region (Kurlansky, 1998). It utilizes a range of marine environments throughout its life, from nearshore habitats as juveniles (e.g., seagrass beds and gravel areas), down to deeper waters on the continental shelf as adults (Bradbury et al., 2008; Petitgas et al., 2013). In the Gullmar Fjord in western Sweden, deemed an important area for marine biodiversity (also a Natura 2000 protected area through the Habitats Directive), Atlantic cod has been partly safeguarded from fishing pressure (January–March) since 2004, which increased to an all year‐round ban from the beginning of 2012 (Länsstyrelsen Västra Götalands Län, 2017). In the shallow, soft‐bottom environments in this protected region, 1‐ to 3‐year‐old Atlantic cod are thought to be the most abundant life stage of cod (Pihl, 1982; Staveley, Perry, Lindborg, & Gullström, 2017; Wennhage & Pihl, 2002), where their prey (e.g., crustaceans, fish) can also be found in high abundance. This local protection promotes survival in the Fjord region, which potentially acts as a source to nearby unprotected populations. This is strengthened by recent evidence that spawning events are highly likely to be occurring in the Gullmar Fjord and other nearby coastal areas in this region (Svedäng et al., 2018). However, there is debate on whether local, genetically separated subpopulations exist in this region or whether they stem from a mix of western Baltic and North Sea Atlantic cod (Cardinale, Mariani, & Hjelm, 2019; Svedäng et al., 2018).

Environmental processes can play an important role in determining the spatiotemporal locations of organisms and individuals (Howey, Wetherbee, Tolentino, & Shivji, 2017; Linderholm et al., 2014; Nilsson, Ogonowski, & Staveley, 2016). Particularly, this concerns those species whose reproductive strategies, development, and movement are directly linked to environmental conditions (Brander, 1995; Freitas, Olsen, Knutsen, Albretsen, & Moland, 2016; Geffen, Fox, & Nash, 2006; Lédée, Heupel, Tobin, Mapleston, & Simpfendorfer, 2016). Atlantic cod, like many cold‐water fish, are physiologically adapted to tolerate a high thermal variation (Metcalfe, Le Quesne, Cheung, & Righton, 2012; Righton et al., 2010), even more so during juvenile stages (Björnsson, Steinarsson, & Oddgeirsson, 2001). Physiological stresses have been much studied (Brander, 1995; Righton et al., 2010), for example, demonstrating how optimal thermal thresholds are required for growth and fecundity. However, exploring how movement behavior connects nursery habitat and how this process is influenced by environmental factors has received much less attention (although see Freitas, Olsen, Moland, Ciannelli, & Knutsen, 2015, Freitas et al., 2016).

Network analysis is currently a frequently used tool for quantifying movement patterns of organisms at multiple spatial scales (Jacoby & Freeman, 2016; Richardson, Giuggioli, Franks, & Sendova‐Franks, 2017; Treml, Halpin, Urban, & Pratson, 2008). Considerable developments have been made in terrestrial ecology using network theory to assess, for example, landscape fragmentation, species‐site attachment, anthropogenic risk, and ecological connectivity (Bodin & Norberg, 2007; Maciejewski & Cumming, 2016; Wittemyer, Keating, Vollrath, & Douglas‐Hamilton, 2017) with recent applications expanding into marine systems (Finn et al., 2014; Jacoby, Croft, & Sims, 2012; Lédée et al., 2016; Stehfest et al., 2013; Williams et al., 2018). Combining network analysis with biotelemetry data, important and novel information can be gleaned on an animal's ecology and the ways in which it connects its environment at a local and “global” (whole network) scale. With regard to seascape ecology, network analysis offers a means to visually represent and quantify the influence of environment features on the movement of animals between locations.

To measure broad‐scale connectivity and understand which linkages and parts of the seascape that are most important for juvenile and subadult Atlantic cod in coastal areas, we use passive acoustic telemetry and network analysis to address the following questions: (a) How do cod link our different monitoring sites and habitats?, (b) Are there structural differences in the movement patterns at different times of year?, and (c) How does connectivity relate to environmental conditions? Together, these questions aim to address how this coastal fjord system is connected ecologically while exploring the importance of different habitats and movement corridors for juvenile and subadult cod residing in a protected nursery area.

## 2 MATERIALS AND METHODS

### 2.1 Study area

**Figure 1:**
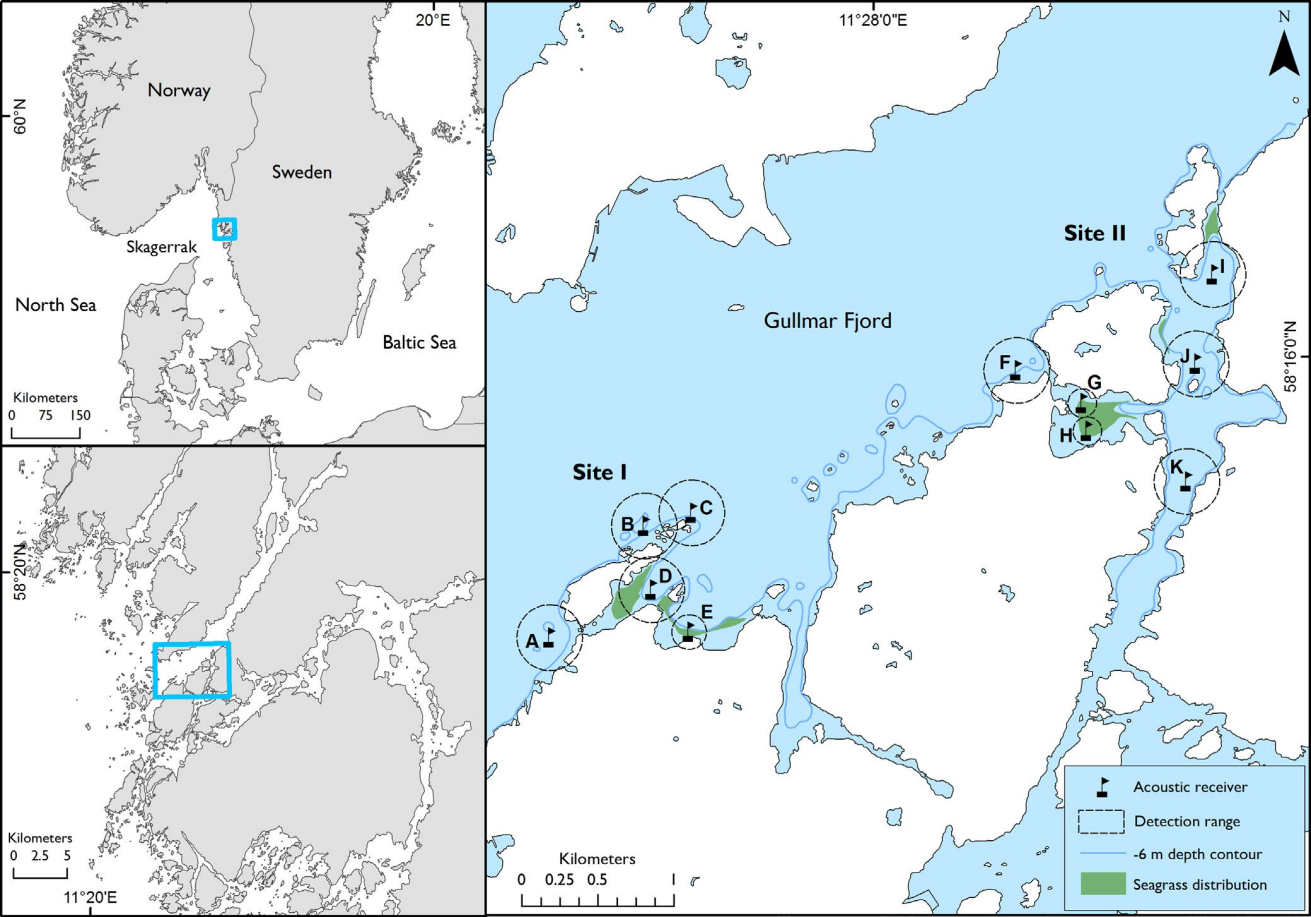
Location of study sites I and II, placement of acoustic receivers (name indicated by letters), receiver detection range, and seagrass habitat distribution (right panel) in the Gullmar Fjord, Sweden. Coastline: ©Lantmäteriet

This study was conducted in two coastal sites (I and II) in the Gullmar Fjord in the Swedish Skagerrak region (Figure 1). This region lies in a transitional area between the Baltic Sea and the North Sea, where tidal influences are low (mean fluctuation < 0.3 m), and the surface salinity is between 24 and 34 (Kristiansen & Aas, 2015). The marine environment is characterized by predominately rocky shores with macroalgae species such as *Fucus* sp. and *Laminaria* sp., and soft sediment bottoms with the presence of seagrass (*Zostera marina*) in the shallower, less exposed areas. In the Gullmar Fjord, shallow bays fringe parts of the shoreline that give way to deeper waters (>60 m), where less exposed bays and channels can be subjected to surface ice coverage during the winter months.

### 2.2 Acoustic telemetry

In order to detect spatial and temporal movement patterns of Atlantic cod (hereafter: cod), an array of 11 acoustic receivers (VR2/VR2W; Vemco) was placed throughout the two sites (Figure 1). Site I is an open and exposed area of coastline with direct access to the deeper fjord waters, while site II is located farther into the fjord and generally more protected with a channel connecting small fjord‐like inlets to the south (Figure 1). Receivers were deployed in the water column, ~1 m above the seafloor using anchors and subsurface buoys, at depths between 2 and 30 m. In order to quantify accurate detection ranges for the receivers, range testing was conducted in both unvegetated (sites I and II) and seagrass‐vegetated (site I) areas. Detection ranges (based on 60% of the detections) were 216 m for unvegetated areas and varied from 94 to 114 m in seagrass (depending on depth; Figure 1). Theoretical detection range overlap occurred between two of the receivers in site I; however, in reality land caused a natural barrier between them. All data were corrected for linear time drift and assessed for false detections (Pincock, 2012) before analyses.

Forty‐eight cod were caught, tagged, and released back into the same sites where they had been captured. The tracking period was from August 2015 to January 2016. Prior to tagging, fish were anesthetised using MS‐222 (0.1 g/L) diluted in seawater, and then measured and weighed. Total length of the cod ranged from 16 to 47 cm (mean total length ± *SD* = 29.8 ± 5.2 cm) with a weight range of 35–569 g (mean weight ± *SD* = 233.8 ± 112 g; Table 1). An incision (~1 cm) was made off center to the mid‐ventral line between the pelvic fins and the anus where an acoustic transmitter (Thelma Biotel, Trondheim, Norway; 7.3 mm diameter, 18 mm length, 1.2 g weight in water, Freq. 69 kHz, 30–90 s. transmitting period; a battery life of 118–185 days) was placed inside the peritoneal cavity. One suture was used to close the incision, and the fish were placed into a recovery tank, with flow‐through seawater, for a maximum of 3 hr before release. The majority of the tagged individuals were estimated to be ~2 years old, thus being immature juveniles (International Council for the Exploration of the Sea, 2004).

**Table 1:** Summary of the 39 fish whose movement patterns were analyzed throughout sites I and II

Transmitter ID	Released site	Weight (g)	TL (cm)	Release date	Days detected	Days monitored	RI	MI
1001	II	356	36	15/08/2015	8	156	0.05	0.25
1002	II	185	26	15/08/2015	71	156	0.46	0.04
1003	II	361	34	15/08/2015	133	156	0.85	0.14
1004	II	225	30	15/08/2015	85	156	0.54	0.14
1006	II	210	28	15/08/2015	27	156	0.17	3.44
1007	II	240	31	15/08/2015	45	156	0.29	0.04
1010	II	209	29	15/08/2015	23	156	0.15	6.39
1011	II	400	37	15/08/2015	94	156	0.60	0.02
1012	II	381	35	16/08/2015	3	155	0.02	16.67
1013	II	264	33	15/08/2015	142	156	0.91	0.06
1014	II	120	25	16/08/2015	143	155	0.92	0.13
1015	II	232	30	16/08/2015	24	155	0.15	0.04
1016	II	422	47	16/08/2015	2	155	0.01	0.50
1017	II	241	30	16/08/2015	88	155	0.57	0.50
1019	II	330	33	16/08/2015	94	155	0.61	0.36
1020	I	136	26	24/08/2015	59	147	0.40	0.03
1021	II	35	16	16/08/2015	5	155	0.03	0.40
1022	II	156	28	16/08/2015	4	155	0.03	8.75
1023	II	150	26	16/08/2015	146	155	0.94	0.56
1024	II	116	25	16/08/2015	61	155	0.39	0.02
1025	I	172	28	24/08/2015	40	147	0.27	0.73
1026	I	228	30	24/08/2015	20	147	0.14	0.20
1027	I	117	25	24/08/2015	128	147	0.87	0.03
1028	I	135	25	22/08/2015	148	149	0.99	0.01
1029	I	194	28	22/08/2015	21	149	0.14	0.38
1030	I	438	37	22/08/2015	99	149	0.66	0.06
1031	I	162	26	22/08/2015	29	149	0.19	0.14
1033	I	249	30	22/08/2015	7	149	0.05	0.86
1034	I	197	29	21/08/2015	109	150	0.73	0.06
1035	I	231	28	21/08/2015	2	150	0.01	0.50
1036	I	569	39	21/08/2015	34	150	0.23	0.88
1038	I	173	27	21/08/2015	149	150	0.99	0.33
1040	I	127	26	16/08/2015	36	155	0.23	2.50
1041	I	205	30	16/08/2015	10	155	0.06	0.40
1042	I	413	36	16/08/2015	12	155	0.08	0.33
1043	I	155	27	16/08/2015	6	155	0.04	0.50
1044	I	266	32	16/08/2015	153	155	0.99	0.05
1045	I	131	25	16/08/2015	20	155	0.13	1.00
1046	I	186	28	16/08/2015	12	155	0.08	0.17

Abbreviations: Days monitored, days from release to end of study; MI, movement index; RI, residency index; TL, total length.

### 2.3 Data analysis

To establish whether differences in movement patterns were present between seasons, data were split into three periods (i.e., summer, autumn, winter), where a distinct change in sea surface temperature (SST) occurred. Summer was considered to be from the 15 August (start of study) to the 5 October 2015, with a mean SST of 16.3°C (±1.8 *SD*); autumn from the 6 October to the 11 December 2015, with a mean SST of 9.4°C (±1.7 *SD*); and winter from the 12 December 2015 to the 18 January 2016 (end of study), with a mean SST of 3.7°C (±2.6 *SD*). In addition to SST, other environmental variables including PAR (photosynthetically active radiation; µmol/m^2^/s), sea level (mm), wind speed (m/s), and wind direction (°) were used as predictors for the networks. All environmental data were grouped into weekly means and sourced from the Sven Lovén Centre for Marine Infrastructure—Kristineberg, University of Gothenburg, with data gathered from Site I.

The initial 24 hr of detections from each individual postrelease were removed from the analysis to eliminate possible irregular movements that may have occurred due to anesthesia and handling stress (Cote, Scruton, Cole, & McKinley, 1999; Knickle & Rose, 2014). In order to determine how long fish were present in the sites, a residency index (RI) was calculated for every individual. This was based on the number of days detected divided by the number of days monitored (i.e., time at liberty; Espinoza, Lédée, Simpfendorfer, Tobin, & Heupel, 2015). Values range from 0 to 1, where individuals near 0 indicate low residency and those close to 1 show high residency.

### 2.4 Network analysis

In order to assess patterns of cod movement in both space and time, network analysis methods were applied to the telemetry data (Jacoby, Brooks, Croft, & Sims, 2012). Receiver nodes within the network were connected by fish movements (edges) indicated by a detection on one receiver followed by the subsequent detection on another receiver. To avoid spatial bias in the network, fish movements that were <4 min between two different receivers were removed, as these were probably due to overlap caused by variation in maximum detection ranges of the receivers. These overlaps were additionally inspected through transmitter/receiver detection graphs to confirm appropriate removal.

Directed, weighted networks were constructed to assess seasonal differences of movements within and between the sites. Firstly, seasonal differences in the relative number of movements (i.e., total number of movements per day divided by the number of fish detected per day) were examined using Welch's *F* test proceeded by a Games–Howell post hoc test to compare differences between seasons. Secondly, overall connectivity was explored by measuring the relative abundance per month of the 16 isomorphism classes (structural variations) of a triad motif sequence for each individual. Motifs are a good measure of substructure within networks and were thus used to quantify shifts in connectivity through time. Motif counts were extracted using the “triad_census” function in the “igraph” package in R (Csardi & Nepusz, 2006). While we recognize the geographic influence of the seascape on limiting the formation of some linkages between specific locations, we take a discrete count‐based approach to motif analysis (Pasquaretta, Jeanson, Andalo, Chittka, & Lihoreau, 2017) to explore the tendency of individuals to alter the frequency of their repeated path use in response to changes in temperature using a negative binomial model (generalized linear mixed model; GLMM).

Undirected, weighted networks were used to measure relationships between environmental factors and node‐based network metrics. To measure and visualize connectivity, network metrics (node strength and degree) were calculated on networks for each individual fish on a weekly (7 days) basis starting from the 25 August 2015 (last date of released fish). Metrics were summed across all receivers for any given week. Node strength is a weighted measure of connectivity defining the cumulative incomings and outgoings per receiver and can be used to explore how often individuals/groups are using certain areas (i.e., hotspots for movement activity). Degree is an unweighted measure of connectivity that gives an indication of how well receivers are connected to each other, which can offer insight into route variability within the constraints imposed by geography.

The above network metrics were analyzed using GLMMs where environmental variables were classed as fixed, and individual fish were classed as random factors. Prior to analyses, environmental predictor variables were transformed as necessary. Wind speed was log‐transformed and PAR square‐root transformed to assume normality. Predictor variables were tested for collinearity using the variance inflation factor (VIF), and those with a score > 3 were not included in the models (i.e., sea level and PAR). Node strength was modeled with a negative binomial distribution (to avoid over‐dispersion) and a log link in relation to (a) SST, (b) wind speed(log), and (c) wind direction. Degree was modeled with zero‐inflated negative binomial distribution and a log link in relation to i) SST, and ii) wind direction.

A movement index (MI) was calculated per individual to give a relative indication of how mobile or stationary each fish was during its time within the array. The numbers of movements were divided by total days detected, where higher values indicated higher mobility. Both weight and total length of fish were tested against RI and MI to establish whether individual size correlated with resident or movement indices. All analyses were performed in R version 3.5.0 (R Development Core Team., 2018) with packages “igraph” (Csardi & Nepusz, 2006), “lme4” (Bates, 2018), and “glmmTMB” (Brooks et al., 2017). Statistical significance for all tests was based on a *p*‐value of <.05.

## 3 RESULTS

From the 48 tagged cod, the 11 receivers recorded 562,502 detections over the 5‐month study period. Three fish left the array during the initial 24 hr, and a further six fish showed no movement patterns, and thus, these were excluded from further analyses. Of the remaining 39 fish, 838 movements were observed over the course of the study (5 months). Residency of individuals varied considerably from 2 to 153 days, giving an average residency index (RI; mean ± *SE*) of 0.38 ± 0.06 (Table 1; Appendix S1). There was no significant difference in RI between sites (*t*‐test, *p* = 0.7). Movement of individuals varied considerably from 1 to 147 observed movements with no significant difference between sites (Wilcoxon test, *p* = 1). No significant relationships (*p*‐value > .05, Spearman's rank correlation) were found between weight and total length against RI and MI, indicating that movement was irrespective of fish size.

**Figure 2:**
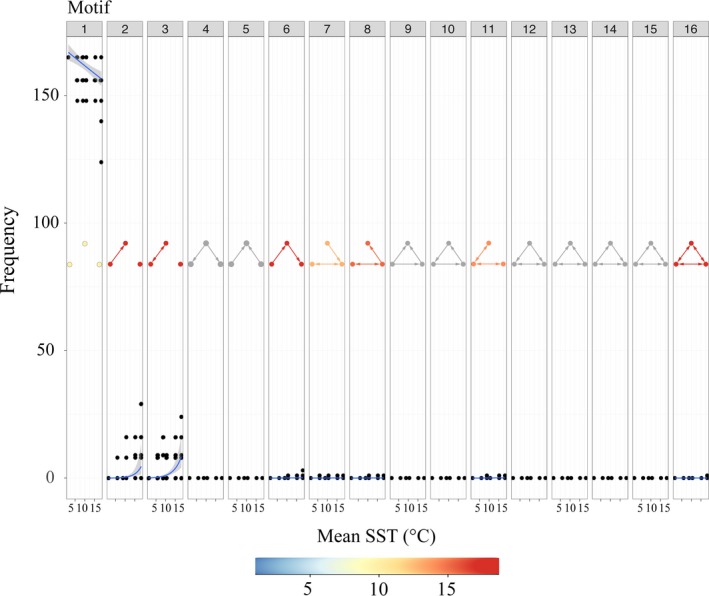
The relationship between connectivity and temperature from the movements of 39 individual cod. Frequencies of all 16 triadic isomorphs from a possible total of 165 motifs (*n* combinations with 11 node networks). Counts were modeled with negative binomial GLMMs where best fitted lines indicate a significant influence of temperature on motif count. GLMM estimates, *z*‐values, and *p*‐values are available in Appendix S2. Isomorphs are colored relative to the temperature scale reflecting the temperature at which the highest count occurred (n.b. temperatures were averaged where joint highest counts occurred and gray isomorphs did not exist in any of the individual movement networks)

Connective redundancy within the movement network was high when considered at the global scale with several of the higher order, more complex motifs nonexistent within any individual movement network. The null triadic motif (i.e., motif 1, no movement) decreased significantly with increasing temperatures, while simple motifs (2 and 3) increased in response (Figure 2). Where higher order motifs did occur, either for an individual or multiple individuals, they tended to occur only when the mean SST was above approximately 12°C (i.e., in the summer months, Figure 2).

**Figure 3:**
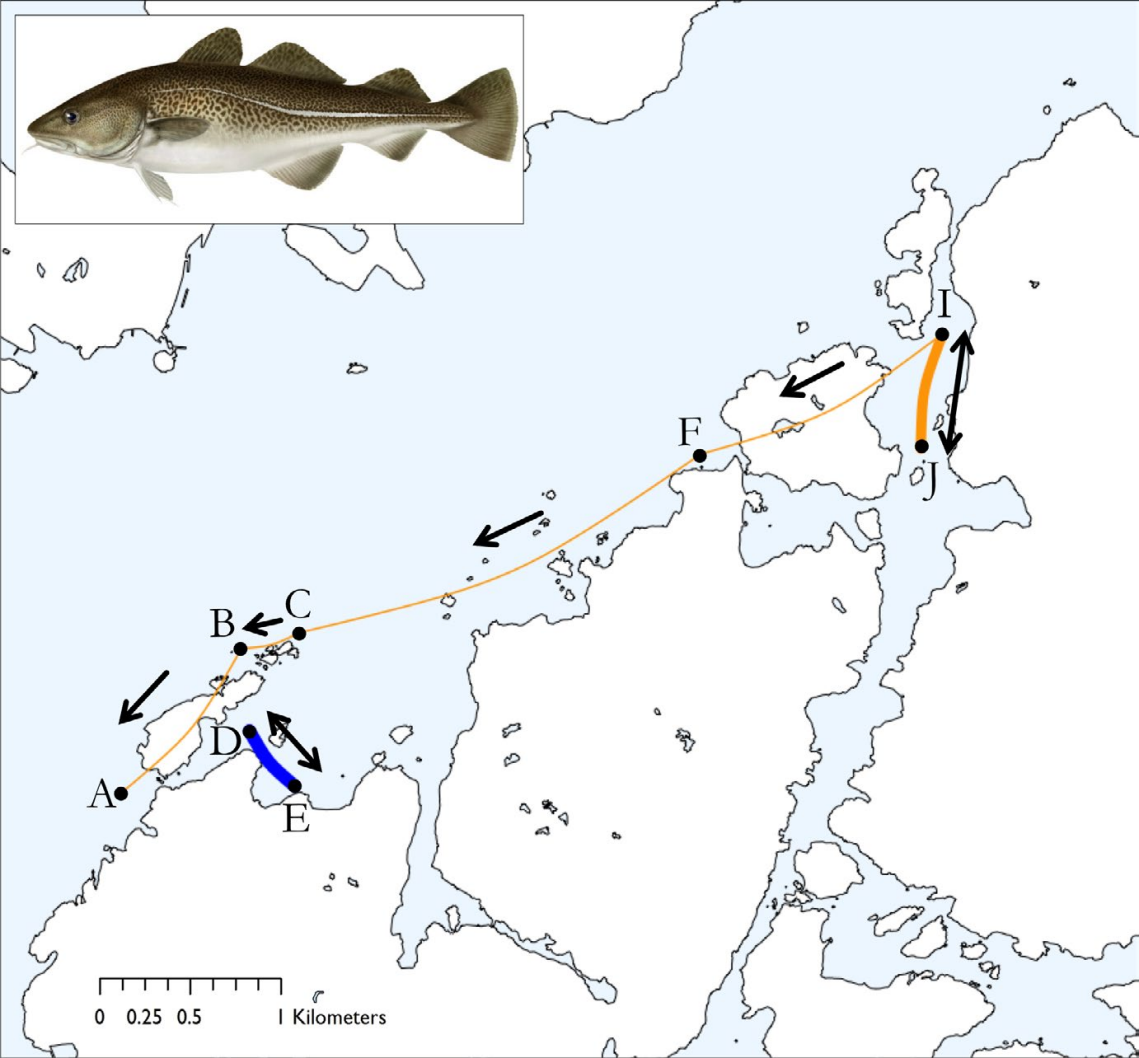
Movement networks of fish #1022 (orange line; connecting A‐J) and #1038 (blue line; connecting D‐E) throughout the study period. Fish #1022 exhibits a low RI and a high MI, whereas fish #1038 displays the opposite with a high RI and a low MI (Table 1). Filled circles marked with letters indicate receiver (node) positions. Colored lines represent movement of fish, where line thickness is relative to the number of movements (range 1–49). Arrows show direction of movement. Top left: Study species Atlantic cod (*Gadus morhua*; Illustration by Karl Jilg, published with permission from the Swedish Species Information Centre (ArtDatabanken), SLU)

The majority of individuals that were observed over a relatively long period (i.e., high RI) showed movement only within their respective site, I or II (e.g., Fish 1038; Figure 3 and Table 1). In contrast, three fish specimens (i.e., No.'s 1022, 1041 and 1042) showed movement patterns connecting the two sites (Figure 3). However, these fish were only detected for a short time, hence showing a low RI (Table 1), indicating that they were in transit before moving to other areas outside the receiver array.

**Figure 4:**
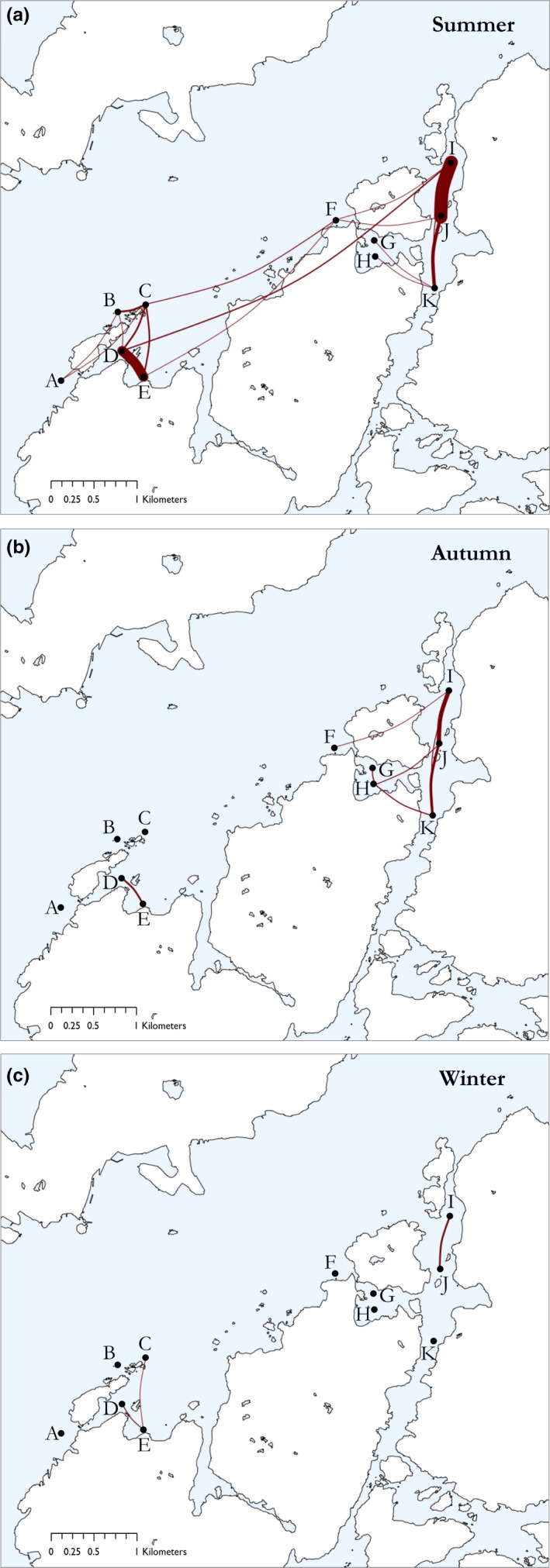
Aggregated seasonal movement networks of juvenile and subadult Atlantic cod (*n* = 39) throughout the study sites in the Gullmar Fjord in (a) summer, (b) autumn, and (c) winter 2015/2016. Letters indicate receiver (node) positions. Red lines represent movement of fish, where line thickness is relative to the number of movements

**Figure 5:**
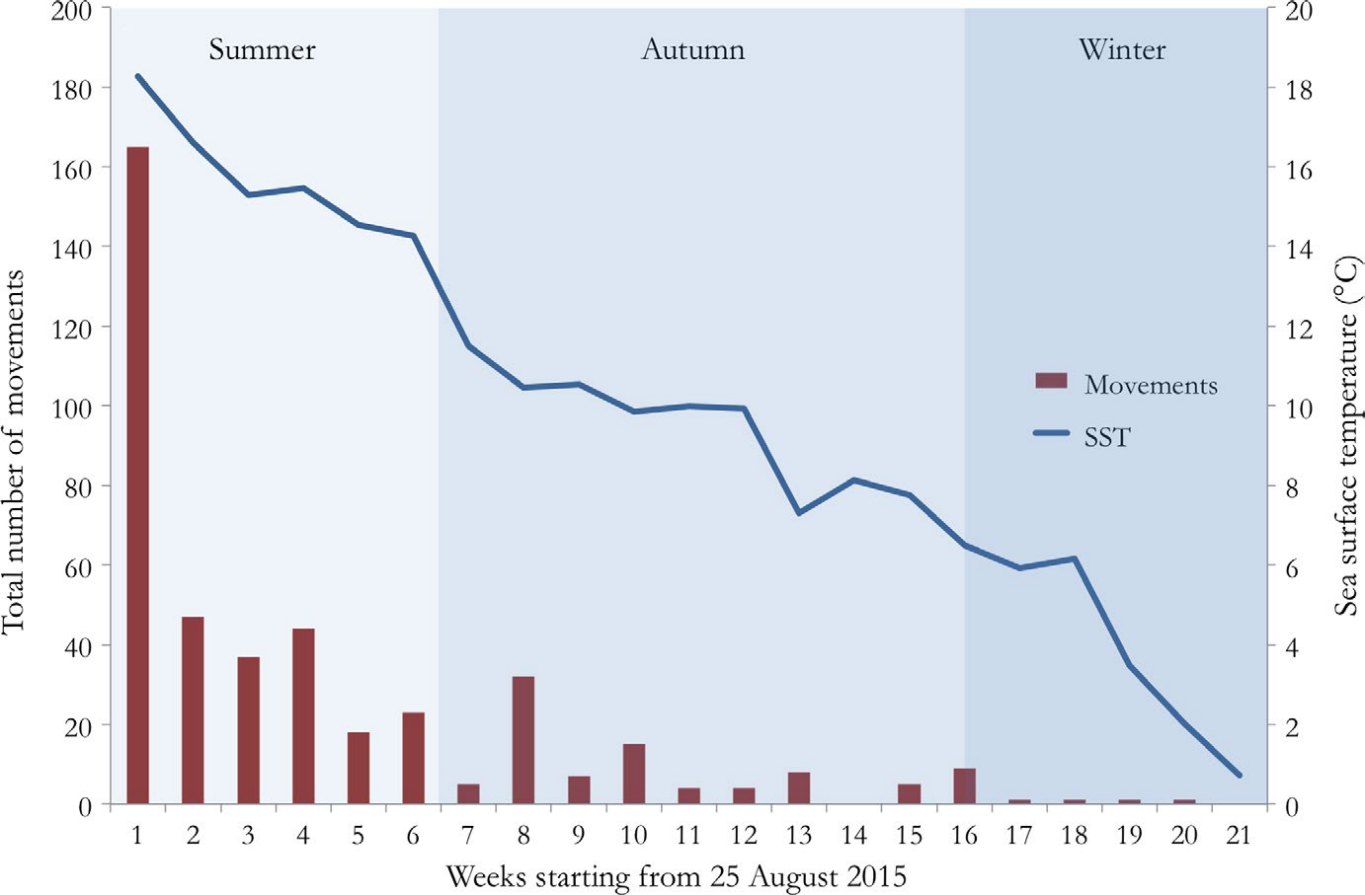
Weekly total number of cod movements and sea surface temperature (SST) means throughout summer, autumn, and winter 2015–2016 starting from the 25 August 2015

The relative number of movements differed significantly between seasons (*F* = 13.36, *p* < .001). A Games–Howell post hoc test comparison revealed that the number of movements in summer was significantly higher than in autumn (*p* = .01) and winter (*p* < .01) and that movements in autumn were higher compared with winter (*p* = .012) (Figures 4 and 5). Movement patterns in the summer revealed more frequent movements between certain receivers, in particular those between D‐E and I‐J. In addition, both sites were connected via fish movement during the summer (Figure 4a). Throughout the autumn and winter, movement activity subsided and connectivity was lost between sites and, to a certain extent, within sites. During these colder months, relatively small‐scale movement patterns were observed (Figure 4b,c).

Sea surface temperature was the only variable that had a significant relationship (positive) with node strength (number of incomings and outgoings) and degree (connectedness of nodes; Table 2). This indicates that temperature plays an important role in movement and connectivity of juvenile and subadult cod.

**Table 2:** Results from the GLMMs showing effects of environmental predictors on cod network metrics (i.e., the responses)

Response	Predictor	Estimate ± *SE*	*z*	*p*
Degree	SST	0.250 ± 0.035	7.107	<.001*
	Wind direction	−0.006 ± 0.003	−1.835	.067
Node strength	SST	0.262 ± 0.034	7.748	<.001*
	Wind speed (log)	1.081 ± 0.916	1.180	.238
	Wind direction	−0.008 ± 0.005	−1.647	.099

Abbreviation: SST, sea surface temperature.

* Significance where *p* < .05.

## 4 DISCUSSION

In this study, we assessed spatial and temporal movement patterns of juvenile and subadult cod that revealed important seasonal changes in connectivity at different scales (100s m to km) throughout a coastal fjord system. We found that a drastic decline in movement and connectivity of juvenile and subadult cod occurred as the water temperature decreased. This was due to many individuals becoming more stationary but also, in part, individuals leaving the study area (Appendix S3) or potentially increased rates of mortality. Further investigation and longer time series would be required to fully determine which response is more likely. Sea temperature is known to have an important influence on organism physiology (Metcalfe et al., 2012), and species diversity and distribution in the marine environment (Day, Stuart‐Smith, Edgar, & Bates, 2018; Tittensor et al., 2010). Movement activity and behavior of fish can be attributed to changes in their external environment, which can show how certain species respond positively to increasing water temperature (Murray, Cowley, Bennett, & Childs, 2018) as well as individual personality differences affecting, for example, home range (Villegas‐Ríos, Réale, Freitas, Moland, & Olsen, 2017, 2018). In contrast, in a temperate fjord in Norway, Freitas et al. (2016) assessed the influence of vertical sea temperature fluctuations on juvenile and adult cod (larger than observed in this study) and found that they were predominantly residing in waters below 16°C, thus shifting their position in the water column to stay within their optimal thermal niche.

We found that cod was present in the study sites while sea surface temperatures were at the extreme ends of their thermal tolerance, resulting in less connectivity within the fjord system during wintertime. This finding is supported by the fact that juveniles possess higher levels of plasma than adults, which acts as an antifreeze in subzero conditions, allowing them to better survive winter conditions in icy coastal areas (Kao & Fletcher, 1988). Indeed, juvenile Atlantic cod may face a trade‐off between increased movement activity and risk of predation or remain stationary and endure the physiological stress associated with seasonal temperature extremes. Interestingly, we found that there was no relationship between residency, or movement, and cod size in this study. However, this could be due to the relatively low variation in size of tagged individuals.

Over time, the number of movements substantially decreased, as did the regularity of route use. This led to a reduction in connectivity between the two sites after the summer but also highlighted certain areas where movement activity occurred throughout the entire study period. Even though most fish clearly became more stationary in their behavior when the season changed toward colder mean temperatures, specific areas (i.e., D‐E and I‐J) seemed to be significant for movement (i.e., localized connectivity) throughout all seasons. Differences in habitat availability (e.g., amount of seagrass) between sites may explain the response of older juveniles utilizing multiple habitats in the shallow‐water seascape. As Pihl et al. (2006) found, habitat choice shifted from the dominance of seagrass during earlier juvenile stages toward using both unvegetated areas and seagrass meadows as they developed. In addition to these habitats, cod may also be using the rocky shoreline (and associated macroalgae) that offers a multitude of nooks and crevices to hide and rest, which incidentally could cause a physical barrier blocking acoustic signals.

In the Skagerrak region, other tagging studies (Rogers, Olsen, Knutsen, & Stenseth, 2014) have shown that cod populations are relatively stationary compared with populations elsewhere in the North Sea. This may be similar in the Gullmar fjord, as some specimens were detected until the end of the study. Perhaps for those fish that were no longer detected by the array, they may have moved to other shallow‐water areas in close proximity, but out of detection, or into deeper fjord waters or simply further offshore (André et al., 2016; Pihl & Ulmestrand, 1993). In Newfoundland, Canada, Cote, Moulton, Frampton, Scruton, and McKinley (2004) found that, during winter, coastal juvenile cod (2–3 years) showed signs of both resident and migratory behavior, indicating individual variation in movement strategies, regardless of being exposed to identical environmental conditions. Further studies with multiyear, high‐resolution tracking of juvenile and subadult Atlantic cod (<30 cm) would be beneficial to be able to explore this variation further. These constantly developing technologies and analyses surrounding animal tracking methods in the marine environment are giving researchers the tools to unravel and understand movement patterns of marine animals at multiple spatial scales.

## 5 CONCLUSION

The use of network analysis, combined with passive acoustic telemetry data, is important for highlighting areas of particular conservation or ecological interest or critical movement pathways for organisms. In this study, investigating movement patterns of juvenile and subadult Atlantic cod, we found a reduction in movement and regularity of routes used as the sea surface temperature decreased from summer to winter. Important shallow‐water areas were identified as particular hotspots for movement activity. Habitats in these hotspot areas varied from dominance of unvegetated soft bottoms to structurally complex seagrass meadows. The findings demonstrate that for the particular life stage studied (i.e., older juveniles and subadults), multiple habitats within the coastal fjord system are important for the survival of local cod populations. This study strengthens the overall understanding of how temperate marine environments are being utilized by juvenile and subadult cod, thereby ascertaining vital information that can be used to improve science‐based resource management, conservation efforts, and fisheries management.

## CONFLICT OF INTERESTS

The authors declare that they have no competing interests.

## AUTHORS' CONTRIBUTIONS

TABS, DMPJ, DP, IL, MC, and MG: conceived the study. TABS, DP, FVDM, IL, MC, and MG: performed fieldwork. TABS, DMPJ, and FVDM: analyzed the data. TABS, DMPJ, DP, FVDM, and MG: contributed to writing the paper. All authors read, commented on, and approved the final manuscript.

## Data Availability

Data deposited in the Dryad Digital Repository: https://doi:10.5061/dryad.m73k28k (Staveley et al., 2019).
