# Supplementary material for: Sea surface temperature dictates movement and habitat connectivity of Atlantic cod in a coastal fjord system
Source: Ecol Evol. 2019 Jul 21;9(16):9076–86. doi: 10.1002/ece3.5453 (PMC6706200; doi:10.1002/ece3.5453)
Supplement: Supplementary file 1 [file ECE3-9-9076-s001.pdf]

Additional file 1

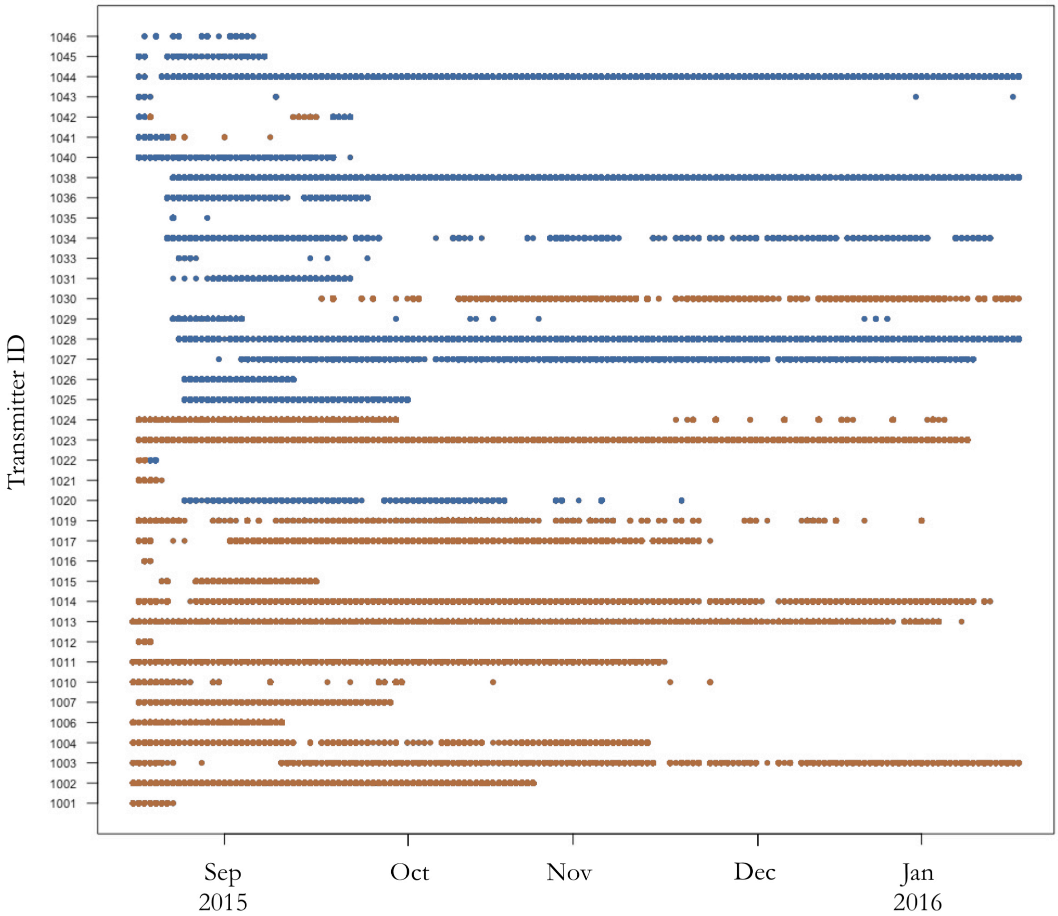

Supplementary Figure 1: Presence plot of Atlantic cod (n = 39) detected in sites I (blue) and II (orange) in the Gullmar Fjord from August 2015 to January 2016. Each filled circle represents one day.
