# Supplementary material for: Sea surface temperature dictates movement and habitat connectivity of Atlantic cod in a coastal fjord system
Source: Ecol Evol. 2019 Jul 21;9(16):9076–86. doi: 10.1002/ece3.5453 (PMC6706200; doi:10.1002/ece3.5453)
Supplement: Supplementary file 2 [file ECE3-9-9076-s002.pdf]

Additional file 2

Supplementary table 1: Output of negative binomial GLMMs frequency of each motif and temperature.

Motif	Estimate (SE)	z-values	P-values
1	-0.042 (0.009)	-4.498	<0.001
2	6.895 (1.476)	4-671	<0.001
3	3.999 (0.007)	536.7	<0.001
6	3.814 (1.376)	2.772	0.005
7	1.425 (0.5682)	2.508	0.012
8	3.456 (0.771)	4.481	<0.001
11	2.984 (0.629)	4.748	<0.001
16	97.955 (3.248)	30.16	<0.001
