# Supplementary material for: Sea surface temperature dictates movement and habitat connectivity of Atlantic cod in a coastal fjord system
Source: Ecol Evol. 2019 Jul 21;9(16):9076–86. doi: 10.1002/ece3.5453 (PMC6706200; doi:10.1002/ece3.5453)
Supplement: Supplementary file 3 [file ECE3-9-9076-s003.pdf]

Additional file 3

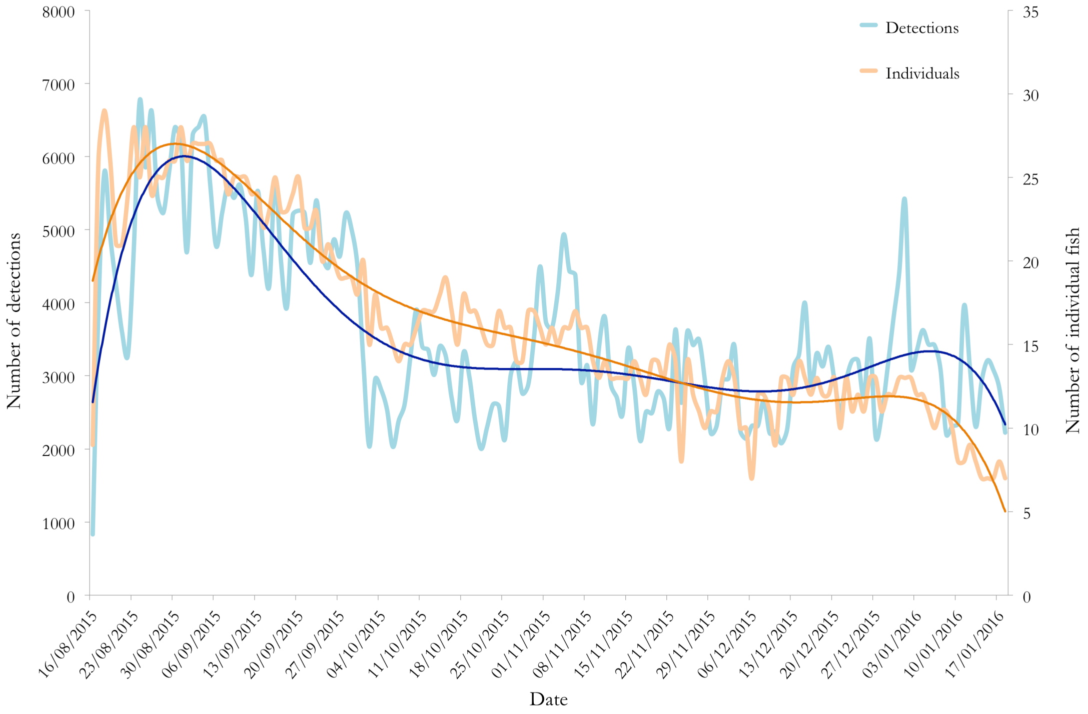

Supplementary Figure 2: Daily total detections and number of individuals detected (n = 45) in sites I and II in the Gullmar Fjord from August 2015 to January 2016. Polynomial trend lines are present for easier visual representation of temporal changes. Note that all tagged fish were released by the 24th of August 2015, which explains the initial low values before this date.
